# New Insights into the DT40 B Cell Receptor Cluster Using a Proteomic Proximity Labeling Assay*[Fn FN2]

**DOI:** 10.1074/jbc.M113.529578

**Published:** 2014-04-04

**Authors:** Xue-Wen Li, Johanna S. Rees, Peng Xue, Hong Zhang, Samir W. Hamaia, Bailey Sanderson, Phillip E. Funk, Richard W. Farndale, Kathryn S. Lilley, Sarah Perrett, Antony P. Jackson

**Affiliations:** From the ‡ National Laboratory of Biomacromolecules, Institute of Biophysics, Chinese Academy of Sciences, 15 Datun Road, Beijing 100101, China,; the ¶ Department of Biochemistry, Tennis Court Road, University of Cambridge, Cambridge CB2 1QW, United Kingdom,; the ‖ Cambridge Centre for Proteomics, Tennis Court Road, University of Cambridge, Cambridge CB2 1QR, United Kingdom,; the ** Department of Biological Sciences, DePaul University, Chicago, Illinois 60604, and; the § University of Chinese Academy of Sciences, 19A Yuquan Road, Beijing 100049, China

**Keywords:** Immunology, Integrin, Lymphocyte, Mass Spectrometry (MS), Proteomics, B Cell Receptor, Evi2a, Raftlin, SILAC, chB6

## Abstract

In the vertebrate immune system, each B-lymphocyte expresses a surface IgM-class B cell receptor (BCR). When cross-linked by antigen or anti-IgM antibody, the BCR accumulates with other proteins into distinct surface clusters that activate cell signaling, division, or apoptosis. However, the molecular composition of these clusters is not well defined. Here we describe a quantitative assay we call selective proteomic proximity labeling using tyramide (SPPLAT). It allows proteins in the immediate vicinity of a target to be selectively biotinylated, and hence isolated for mass spectrometry analysis. Using the chicken B cell line DT40 as a model, we use SPPLAT to provide the first proteomic analysis of any BCR cluster using proximity labeling. We detect known components of the BCR cluster, including integrins, together with proteins not previously thought to be BCR-associated. In particular, we identify the chicken B-lymphocyte allotypic marker chB6. We show that chB6 moves to within about 30–40 nm of the BCR following BCR cross-linking, and we show that cross-linking chB6 activates cell binding to integrin substrates laminin and gelatin. Our work provides new insights into the nature and composition of the BCR cluster, and confirms SPPLAT as a useful research tool in molecular and cellular proteomics.

## Teaser

**Background:** B cell receptor (BCR) clusters modulate BCR signaling in B-lymphocytes.

**Results:** We used a quantitative proteomic proximity assay to analyze the BCR cluster in DT40 cells.

**Conclusion:** Our proximity labeling assay identified novel components of the BCR cluster linked to integrin signaling.

**Significance:** We provide new insights into BCR assembly and identify new and unexpected targets for further functional analysis.

## Introduction

The B cell receptor (BCR)^6^ is an IgM class immunoglobulin present on the plasma membrane of B-lymphocytes. When either soluble or membrane-bound antigen binds to the BCR it becomes cross-linked, and assembles into spatially restricted and functionally integrated protein clusters (1). The cross-linked BCRs enter lipid rafts, and drive their coalescence (2). These events activate a complex series of signal transduction pathways, followed by the “capping” and endocytosis of the BCR-antigen complex (3). As a consequence, the B-lymphocyte can either undergo division and differentiation, ultimately to produce an antibody-secreting plasma cell, or can initiate apoptosis. Which pathway is followed depends on the developmental stage of the B-lymphocyte and its immunological context (4).

The molecular composition of the BCR cluster is of considerable interest. Currently, however, it is not well characterized, and the proteomic analysis of such clusters represents a significant technical challenge (5). This is in part because the BCR clusters are highly dynamic, and their composition may change during the BCR cross-linking process (1, 6). More fundamentally, proteins that become concentrated in these clusters may not necessarily bind each other directly, or may bind only weakly. Traditional isolation methods such as immunoprecipitation may not be effective in this case. Although cross-linking could theoretically be used, in practice it can be difficult to control (7).

We believe that alternative analytical methods will be required to better survey protein diversity within membrane-localized structures such as the BCR cluster. Recently, several groups have described the use of “proximity labeling assays” for the investigation of membrane proteins (8, 9). In these methods, an appropriately targeted peroxidase enzyme acts on a substrate to generate a short-lived product capable of covalently labeling proteins in the immediate vicinity of the enzyme. The labeled proteins can then be isolated by standard affinity chromatography, and identified by mass spectrometry (MS).

Here we describe a new variation of proximity labeling, which combines cleavable tyramide-biotin chemistry with high throughput proteomics, made quantitative using stable isotope labeling by amino acids in cell culture (SILAC). We use the method to provide the first proteomic analysis of a cross-linked BCR cluster on any B-lymphocyte. For our model, we use the avian leukosis virus-transformed chicken cell line DT40 (10). This cell line is derived from an immature B-lymphocyte (11). The DT40 cell line is widely used for functional studies, because its high rate of homologous recombination greatly facilitates gene targeting (10). The genes for several proteins within the BCR signaling pathway have been knocked out in DT40 and the functional consequences investigated (12). DT40 cells can also be grown in large numbers, aiding proteomic analysis (13). DT40 cells therefore represent an attractive and physiologically relevant model system on which to apply our proximity labeling assay.

We identify known and suspected components of the BCR clusters, including the lipid raft marker raftlin (14) and integrins (15). We also detect proteins not previously linked to BCR cluster assembly. Surprisingly, we detect protein chB6, a molecule long used as an important allotypic marker for avian B-lymphocyte development, but whose function is currently unknown (16). We show that chB6 is partitioned between lipid rafts and non-raft regions of the DT40 plasma membrane, and we show that BCR clustering drives the close association of chB6 with the BCR. Analysis of the chB6 primary sequence suggests structural similarities to immunoglobulin (Ig) domain-containing signaling and cell-adhesion molecules. Consistent with this view, we show that chB6 cross-linking activates integrin-mediated cell binding. Our results confirm the potential of proximity labeling assays for the analysis of the BCR cluster, and identify new targets for further functional studies.

## EXPERIMENTAL PROCEDURES

### 

#### 

##### Buffers

The composition of buffers used in the biotinylation, purification, and immunofluorescence experiments were as follows: tyramide labeling buffer (50 mm Tris-HCl, pH 7.4, with fresh 0.03% H_2_O_2_ containing 80 μg/ml of tyramide-biotin label), antibody strip buffer (50 mm glycine, pH 3.0, 150 mm NaCl, 0.9 mm CaCl_2_, 0.5 mm MgCl_2_), cell lysis buffer (20 mm Tris-HCl, pH 7.5, 5 mm EDTA, 1× protease inhibitor mixture (Roche Applied Science), 150 mm NaCl, 1% (v/v) Triton X-100, 0.1 m sodium thiocyanate), wash buffer 1 (10 mm Tris, pH 7.4, 1% (v/v) Triton X-100, 1 mm EDTA, 0.5% (w/v) SDS, 500 mm NaCl, 1× protease inhibitor mixture, 0.1 mg/ml of PMSF (Sigma), 0.1 m sodium thiocyanate), wash buffer 2 (0.1 m sodium thiocyanate, 10 mm Tris, pH 7.4, 1% (v/v) Triton X-100, 1 mm EDTA, 0.5% (w/v) SDS, 1× protease inhibitor, 0.1 mg/ml of PMSF), elution buffer (0.1 m sodium thiocyanate, 1% (w/v) SDS, 5 mm TCEP (Thermo), 100 mm Tris, pH 7.4, 1× protease inhibitor mixture, 0.1 mg/ml of PMSF), immunofluorescence wash buffer (PBS containing 0.5% saponin, 0.1% Triton X-100), immunofluorescence blocking buffer (immunofluorescence wash buffer containing 10% non-immune goat serum), immunofluorescence reducing buffer (5 mm TCEP in 150 mm NaCl, 1 mm EDTA, 0.2% BSA, 20 mm Tris, pH 8.6) (17), and TBS-Tween (25 mm Tris, 150 mm NaCl, 0.05% Tween 20, pH 7.5).

##### Tyramide-biotin Label Preparation

The cleavable tyramide-biotin label was prepared by gently mixing 5 mg of EZ-Link NHS-SS-biotin (Thermo) with 1.55 mg of tyramine hydrochloride (Sigma) in 2 ml of 50 mm borate, pH 8.8, overnight, at room temperature in the dark (18). After filtering with a 0.2-μm filter the label was stored at −20 °C.

##### Cell Culture

Routine growth and maintenance of the DT40 cell line was as previously described (13). For SILAC experiments, RPMI 1640 heavy medium contained ^13^C_6_-labeled l-lysine and l-arginine (K_6_R_6_), whereas the light medium was RPMI 1640 containing non-labeled l-lysine and l-arginine (K_0_R_0_), both of which were supplemented with 10% dialyzed fetal bovine serum (all from Dundee Cell Products), 1% dialyzed chicken serum (in house), 2 mm l-glutamine, 100 units/ml of penicillin, and 100 μg/ml of streptomycin (all from Invitrogen).

##### Tyramide-biotin Labeling

Exponentially growing cells were pelleted and washed in phosphate-buffered saline (PBS) at room temperature. About 5 × 10^8^ cells were incubated with end over end rotation for 2 h with 20 μg/ml of HRP-conjugated goat anti-chicken IgM (Bethyl Ltd.), or 20 μg/ml of HRP-conjugated goat anti-rabbit IgG (Bio-Rad) in 20 ml of PBS with 10% (v/v) goat serum (Ultraclone Ltd.) at 4 °C. Cells were pelleted and re-suspended in 10 ml of tyramide-labeling buffer and incubated with end over end rotation at room temperature for 5 min. After incubation, 100 units/ml of catalase (Sigma) was added and incubated with the samples for a further 5 min to quench H_2_O_2_. Cells were washed gently with 45 ml of antibody strip buffer and left on ice for 5 min. Cells were re-suspended in 1 ml of cell lysis buffer and incubated for 30 min on ice. Insoluble material was removed by centrifugation for 4 min at 130,000 × *g* and protein-containing soluble fraction was recovered for streptavidin-bead capture.

##### Affinity Purification of Biotinylated Proteins

The same amount of specifically and nonspecifically biotinylated proteins (1.04 mg (1st SILAC) and 0.78 mg (2nd SILAC)) were mixed and incubated with 0.25 ml of streptavidin-agarose beads in 1 ml of cell lysis buffer. The beads were washed twice with 1 ml of ice-cold wash buffer containing sodium thiocyanate to reduce nonspecific interactions (19). Beads were then incubated in 1 ml of elution buffer with rotation for 1 h at 4 °C. The biotinylated proteins were recovered by centrifugation and concentrated by vacuum drying. Proteins were separated by SDS-PAGE (10%). The gels were stained with colloidal Coomassie Blue prior to dividing and excising into four equal strips. Gel slices were destained in ddH_2_O and 20 mm NH_4_HCO_3_, reduced with 2 mm DTT, and alkylated with 10 mm iodoacetamide prior to overnight digestion with 2 μg of sequencing grade trypsin (Promega). Peptides were extracted with acetonitrile and 1% formic acid and re-suspended in water with 1% formic acid after vacuum drying.

##### Immunoaffinity Purification of the BCR

5 × 10^8^ cells were separately incubated with either HRP-conjugated goat anti-chicken IgM or HRP-conjugated goat anti-rabbit IgG control, lysed as described above. About 1.7 mg of cell lysate protein was incubated with 0.25 ml of bead slurry of rabbit anti-goat IgG-Sepharose (Sigma) overnight at 4 °C, washed extensively with cold PBS, and protein was eluted with 1 ml of 0.1 m glycine, pH 2.5, at 4 °C. Samples were concentrated by freeze drying and separated by SDS-PAGE (4–12% gradient gels). Four equal sized bands were excised and processed for trypsin digestion as above.

##### Liquid Chromatography-Mass Spectrometry

All LC-MS/MS SPPLAT experiments were performed using a nanoAcquity UPLC system (Waters Corp., Milford, MA) and an LTQ Orbitrap Velos hybrid ion trap mass spectrometer (Thermo Scientific, Waltham, MA). Separation of peptides was performed by reverse-phase chromatography using a Waters reverse-phase nanocolumn (BEH C18, 75 μm inner diameter × 250 mm, 1.7-μm particle size) at a flow rate of 300 nl/min. Peptides were initially loaded onto a pre-column (Waters UPLC Trap Symmetry C18, 180 μm inner diameter × 20 mm, 5-μm particle size) from the nanoAcquity sample manager with 0.1% formic acid for 3 min at a flow rate of 10 μl/min. After this period, the column valve was switched to allow the elution of peptides from the pre-column onto the analytical column where a linear gradient of increasing acetonitrile (5–35%) over 60 min was employed. The LC eluant was sprayed into the mass spectrometer by means of a nanospray source. All *m*/*z* values of eluting ions were measured in the Orbitrap Velos mass analyzer, set at a resolution of 30,000. Data-dependent scans (top 10) were employed to automatically isolate and generate fragment ions by collision-induced dissociation in the linear ion trap, resulting in the generation of MS/MS spectra. Ions with charge states of 2+ and above were selected for fragmentation. Post-run, the data were processed using Protein Discoverer (version 1.2, Thermo Scientific).

##### SILAC Data Analysis

The raw MS data files were converted to mgf files and searched against the UniprotKB *Gallus gallus* database (2012, 27,000 entries) using the Mascot search algorithm (version 2.2.07, Matrix Science, London, UK) with methionine oxidation (M) as a variable modification and cysteine carbamidomethylation (C) as a fixed modification, allowing 2 missed cleavages, a peptide mass tolerance of ±1 Da, and a fragment mass tolerance of 0.8 Da. Quantitation was performed using MaxQuant (version 1.0.3.5). Raw data were searched using Andromeda (20), with Arg-6 and Lys-6 set as heavy labels, methionine oxidation and *N*-acetylation as variable modifications, and cysteine carbamidomethylation as a fixed modification. The proteins were considered to be identified if there was at least one unique peptide and to be quantified if there were at least two unique peptides. Only unique peptides were used for quantitation.

##### BCR Immunoprecipitation

LC-MS/MS experiments were performed as above but using a nanoLC Ultra 1D plus system (Eksigent Corp., Dublin, CA) and an LTQ Orbitrap at a resolution of 60,000. Peptides were initially loaded onto an in-house pre-column (100 μm inner diameter × 40 mm, 3.6-μm particle size) from a nanoLC AS-2 autosampler with 0.5% formic acid at a flow rate of 1.5 μl/min, then further separated using an in-house reverse-phase nanocolumn (Phenomenex, kinetex C18, 75 μm inner diameter × 150 mm, 2.6-μm particle size) at a flow rate of 250 nl/min for 120 min. Peptide mass tolerance was ±20 ppm and fragment mass tolerance was 0.8 Da. Significance threshold was set to *p* > 0.05 and ion score cut off was set to 0. Protein abundance was estimated by calculating emPAI scores (21).

##### Immunoprecipitation of Selected Biotinylated Proteins

Tyramide-biotin labeling of about 5 × 10^8^ cells was carried out as described above. Cells were lysed in 0.5 ml of lysis buffer without sodium thiocyanate for 30 min at 4 °C. Insoluble material was removed by brief centrifugation (2000 × *g*, 3 min). Lysate supernatants were separately incubated with mouse monoclonal anti-CDC42 (Abcam ab170048) (1:100 dilution) or mouse FU5–11G2 monoclonal anti-chB6 (1:100 ascites (22)) overnight at 4 °C. Samples were then incubated with 0.05 ml of slurry of protein G-Sepharose (Sigma) with end over end rotation for 2 h at 4 °C and pelleted by centrifugation (2000 × *g*, 3 min). To remove weakly interacting proteins, pellets were washed four times in 1.5 ml of ice-cold lysis buffer and four times in 1.5 ml of ice-cold lysis buffer containing 0.75 m NaCl all without sodium thiocyanate, with 5-min incubations between spins. The immunoprecipitated proteins were then eluted with 0.08 ml of antibody strip buffer at 50 °C for 5 min. The resulting supernatants were separated by SDS-PAGE (10%) without reducing agent and electrophoretically transferred onto a PVDF membrane using the iBlot gel transfer system (Invitrogen). Separate blotted samples were blocked for 3 h at 4 °C with 4% BSA in TBS-Tween, then probed overnight at 4 °C with affinity-purified rabbit anti-chB6 specific peptide EMIADVESQENASNC (Dragonfly Sciences, Wellesley, MA) (1:200 dilution in TBS-Tween), or polyclonal rabbit anti-CDC42 (Abcam ab175270) (1:100 dilution in TBS-Tween). Membranes were washed with TBS-Tween, then probed with HRP-conjugated goat anti-rabbit (Dako) at 1:5000 dilution in TBS-Tween for 1 h at room temperature. The membranes were then washed with TBS-Tween four times for 5 min each at room temperature. Bands were visualized by enhanced chemiluminescence (Pierce). In parallel, separate blots were incubated with HRP-conjugated streptavidin (Molecular Probes) (1:8000 dilution in TBS-Tween) for 1 h at 4 °C to detect biotinylation. Blots were washed as above, and bands were visualized by enhanced chemiluminescence (Pierce).

##### Co-localization of BCR and Deposited Tyramide-biotin

DT40 cells were incubated with HRP-conjugated goat anti-chicken IgM, or HRP-conjugated goat anti-rabbit IgG and tyramide biotinylated as described above. Cells were attached to coverslips pre-coated with 0.05% poly-l-lysine and fixed with 4% paraformaldehyde in PBS for 10 min at room temperature as described previously (13). Cells were washed four times in immunofluorescence wash buffer followed by immunofluorescence blocking buffer for 1 h at room temperature. Cells were stained with FITC-conjugated donkey anti-goat IgG (1:100) and Alexa 568-conjugated avidin (1:2000) (both Abcam) in blocking buffer for 1 h at room temperature. Cells were washed four times, and coverslips were mounted with Vectashield (Vector) and examined using an Olympus Fluoview IX81 laser scanning confocal microscope. To test for surface biotinylation, cells were preincubated with HRP-conjugated anti-chicken IgM, tyramide-biotinylated as described above, then half the cell population was incubated with immunofluorescence reducing buffer for 10 min. Cells were stained with FITC-conjugated anti-goat IgG to detect BCR and Texas Red-conjugated avidin to detect the remaining biotin. Images were rendered in pseudo-three-dimensional using ImageJ software (National Institutes of Health; rsbweb.nih.gov/ij).

##### Immunofluorescence

For integrin and BCR immunofluorescence, cells were preincubated with HRP-conjugated anti-chicken IgM, fixed, permeabilized, and incubated with FITC-conjugated donkey anti-goat IgG as above for 1 h. Cells were washed six times in immunofluorescence wash buffer. Cells were incubated with mouse anti-chicken β1 integrin subunit (1:100) (Developmental Studies Hybridoma Bank) for 1 h in immunofluorescence wash buffer. Cells were washed six times with immunofluorescence blocking buffer, followed by staining with Alexa 568-conjugated anti-mouse IgG (1:100) (Sigma) in immunofluorescence blocking buffer. Cells were processed and analyzed for fluorescence microscopy as above. Control cells lacking either HRP-conjugated anti-IgM or primary antibodies were identically processed.

For co-localization of chB6 and lipid rafts, ∼10^6^ cells were suspended with primary antibody FU5-11G2 (mouse anti-chB6.2 ascites (22)), diluted 1:100, and incubated for 20 min on ice. The cells were then washed twice and resuspended with FITC-conjugated anti-mouse IgG secondary antibody, diluted 1:200 (Millipore, Temecula, CA), and Alexa 568-conjugated cholera toxin subunit B (Invitrogen) diluted 1:800. The cells were then incubated for 20 min on ice. The cells were washed twice and then resuspended in PBS. Images of chB6 immunofluorescence and lipid raft overlay were acquired on an epifluorescence microscope (Nikon Eclipse E600) with ×40 objective. Images were processed with SPOT Advanced software (Diagnostic Instruments, Inc., Sterling Heights, MI).

##### Lipid Raft Isolation and Western Blot Analysis of Membrane Fractions

Detergent-soluble and -insoluble membrane fractions were prepared according to the method of Xie *et al.* (23). Briefly, 2 × 10^7^ DT40 cells were washed and the cell pellet was resolubilized in 1% Brij 58 lysis buffer (1% Brij 58, 20 mm Tris, pH 7.5, 150 mm NaCl, with protease and phosphatase inhibitors). Lysis was allowed to proceed for 30 min on ice. The cell lysate was then centrifuged at 14,000 × *g* for 25 min at 4 °C to separate Brij-soluble and -insoluble fractions. The supernatant (Brij detergent-soluble or non-lipid raft fraction) was kept on ice until later use. The detergent-insoluble pellet (lipid rafts) was resolubilized in octyl glucopyranoside lysis buffer (60 mm octyl glucopyranoside 292, 150 mm NaCl, 20 mm Tris, pH 7.5, 50 mm β-glycerophosphate, 1% Triton X-100, 1% SDS) by sonication, followed by incubation on ice for 30 min. The detergent-insoluble fraction was centrifuged at 14,000 × *g* for 10 min at 4 °C to remove further insoluble material. The supernatant was kept on ice until further use.

Aliquots of detergent-soluble and detergent-insoluble fractions equivalent to 10^6^ cells were resolved on a 10% SDS-PAGE gel with 0.35 m 2-mercaptoethanol, and transferred to nitrocellulose membranes (Micron Separations, Westborough, MA). Membranes were blocked overnight with 4% BSA in TBS at room temperature and then washed twice with TBS-Tween. Membranes were probed with the rabbit anti-chB6 as described above (1:200 dilution in TBS-Tween) overnight at room temperature. Membranes were washed with TBS-Tween and then probed with HRP-conjugated goat anti-rabbit antisera (Pierce) at a 1:5000 dilution for 1.5 h at room temperature. The membranes were washed with TBS-Tween three times for 5 min. Bands were visualized by chemiluminescence (Supersignal, Pierce Chemical) and photographed with a digital imaging system (AlphaInnotech, San Leandro, CA).

##### Mitochondrial Staining

Cells were incubated with HRP-conjugated goat anti-chicken IgM as above. Cells were stained for mitochondria using MitoTracker, diluted to 250 nm in serum- free media (Molecular Probes), following the manufacturer's instructions. Cells were fixed, permeabilized, and incubated with FITC-conjugated donkey anti-goat IgG (1:100), processed, and analyzed for fluorescence microscopy as above.

##### Proximity Ligation Assays (PLA)

DT40 cells were incubated with HRP-conjugated goat anti-chicken IgM as described above. Cells were attached to coverslips pre-coated with 0.05% poly-l-lysine and fixed with 4% paraformaldehyde in PBS for 10 min at room temperature. In the controls, DT40 cells were fixed before incubating with HRP-conjugated goat anti-chicken IgM. Cells were washed six times in immunofluorescence wash buffer followed by immunofluorescence blocking buffer for 1 h at room temperature. Cells were incubated with rabbit anti-chicken raftlin (1:100) (14) or mouse BoA1 monoclonal anti-chB6 (1:100) (24) in blocking buffer for 1 h at room temperature. Cells were processed according to the Duolink PLA instructions, and coverslips were mounted with DAPI mounting solution (Duolink) and examined using an Olympus Fluoview 1000 laser scanning confocal microscope. Quantification of PLA images was performed using the software BlobFinder (25).

##### Multiplex RT-PCR for Integrin α-Subunit Isoforms

Total RNA was extracted from ∼5 × 10^6^ DT40 cells using an RNA extraction kit (Qiagen). RT-PCR was carried out with 1 μg of RNA, using the One-Step RT-PCR kit (Qiagen), following the manufacturer's instructions. The amplification primers used were as follows: chicken integrin α3, forward, 5′-TCTTCGGCTTCTCCGTAGC-3′ (146–164), reverse, 5′-ACTCAGCAGG TACACAGCCC-3′ (824–843); chicken integrin α4, forward, 5′-ATTATAATGGGAGCCCCTGG-3′ (636–665), reverse, 5′-CCCACTGAGCTCTATGTCCA-3′ (1063–1082); and chicken actin, forward, 5′-AACCCCAAAGCCAACAGAG-3′ (399–417), reverse, 5′-TGAGGTAGTCCGTCAGGT-3′ (619–637).

##### Cell Binding to Integrin Substrates

Aliquots of DT40 cells were incubated as above with 20 μg/ml of HRP-conjugated goat anti-chicken IgM, 20 μg/ml of nonspecific goat IgG, mouse monoclonal FU511G2 anti-chB6 (1:100 dilution of ascites), or nonspecific mouse IgG (1:100 dilution of ascites). Cell binding to integrin substrates was monitored using the SRU Biosystems BIND Explorer system. This biosensor measures the shift in peak wavelength (ΔPWV) of light reflected from the base of a well following cell binding after 2 h of adhesion. All experiments were performed in triplicate in the presence of either 5 mm Mg^2+^ or EDTA. Cells were seeded at 2 × 10^5^ cells per well in a 96-well TiO Bind biosensor pre-coated with either 20 μg/ml of laminin, 20 μg/ml of gelatin, or 5% albumin, as previously described (26). Data are presented as the mean ± S.E. and were analyzed for statistical significance using unpaired *t* test with the Prism software package (vs5.0d, GraphPad Software, San Diego, CA).

## RESULTS

### 

#### 

##### Selective Proteomic Proximity Labeling Assay Using Tyramide (SPPLAT)

**FIGURE 1.**
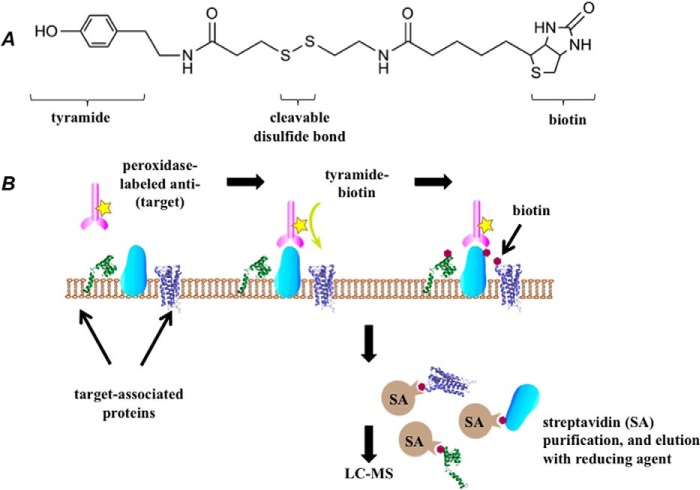
**Outline of the SPPLAT protocol.** *A*, structure of the biotin-tyramide proximity labeling reagent. *B*, principle of the method. The antibody-directed targeting of HRP to a surface protein of interest, followed by brief labeling with biotin-tyramide enables proteins in the immediate vicinity of the target to be biotinylated. These are isolated by incubation of the cell lysate with streptavidin-agarose (*SA*), and elution with reducing agent.

Bivalent antibody against IgM can be used to induce BCR cross-linking and signal transduction (12). We used an HRP-conjugated anti-chicken IgM for this purpose. As the labeling reagent, we used a molecule that connects tyramide to biotin via a spacer arm containing a disulfide bond (Fig. 1*A*). The SPPLAT methodology is outlined in its general form in Fig. 1*B*. In the brief presence of peroxidase (*i.e.* HRP) and hydrogen peroxide, tyramide moiety is converted into a free radical that only diffuses a short distance before covalently labeling protein residues such as tyrosines (27). After cell lysis and capture by immobilized streptavidin, the biotinylated proteins were eluted with reducing agent and analyzed by liquid chromatography-mass spectrometry (LC-MS).

**FIGURE 2.**
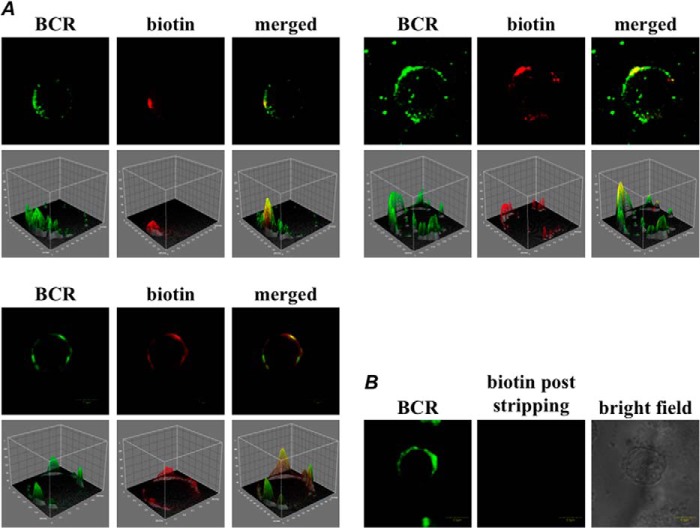
**SPPLAT analysis of the BCR-induced clusters in DT40 cells.** *A*, co-localization of BCR and deposited biotin. Cells were preincubated with HRP-conjugated anti-chicken IgM and tyramide biotinylated. The distribution of BCR and deposited biotin was detected using FITC-conjugated anti-goat IgG and Alexa 568-labeled avidin as described under “Experimental Procedures.” Three representative images are shown with corresponding images rendered as pseudo-three-dimensional. *Bar* = 5 μm. *B*, tyramide-biotinylated cells were stripped of biotin by treatment with impermeant reducing agent as described under “Experimental Procedures.” Cells were stained with FITC-conjugated anti-goat IgG to detect BCR, and with Texas Red-conjugated avidin to detect remaining biotin. *Bar* = 5 μm.

To initiate the tyramide biotinylation, the BCR cross-linking was followed by a 5-min incubation with tyramide-biotin at room temperature. Under these conditions, and consistent with previous reports (28), the cross-linked BCR assembled into spatially restricted membrane patches. Immunolocalization confirmed that the surface distribution of the BCR and the deposited biotin were closely correlated (Fig. 2*A*). Furthermore, most of the biotin could be removed by mild reduction with the membrane-impermeant reducing agent TCEP (17) (Fig. 2*B*). Hence, the biotin predominantly labeled the extracellular face of the plasma membrane, in close proximity to the BCR. MS analysis confirmed the BCR was immunoprecipitated by the anti-chicken IgM under these conditions (supplemental Table S1).

**FIGURE 3.**
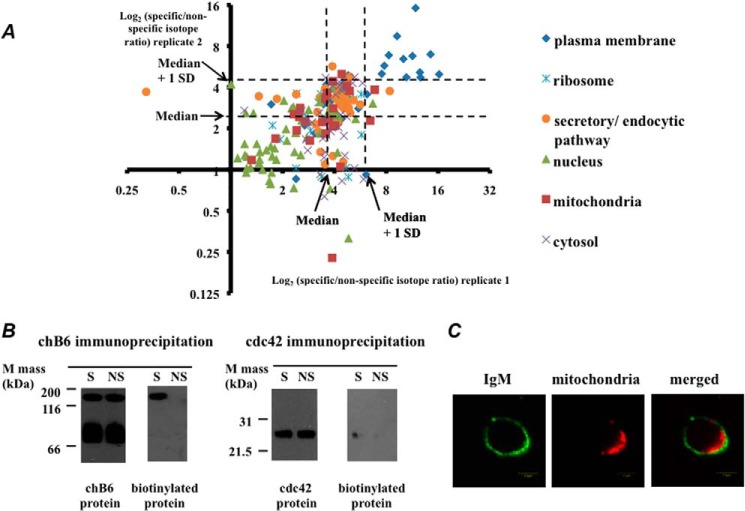
**Summary of quantitative SILAC data.** *A,* scatter plot showing the isotope ratios for each protein quantitatively identified in both independent SILAC experiments. The organelle locations of the proteins are indicated. *Broken lines* represent the median value plus 1 S.D. for each data set. *B,* proteins chB6 and CDC42 were immunoprecipitated from specifically labeled (*S*) and nonspecifically labeled cells (*NS*), then separately probed with antibodies against chB6 or CDC42 and with HRP-streptavidin to detect biotin labeling as indicated. These samples were run under non-reducing conditions. *C*, immunofluorescence image showing accumulation of mitochondria underneath the BCR surface cluster. Mitochondria were detected using MitoTracker staining, and BCR was detected by immunofluorescence as described under “Experimental Procedures.” *Bar* = 5 μm.

We used SILAC to provide a quantitative protocol (29). Here, DT40 cells were grown in medium containing either heavy (^13^C) or light (^12^C) l-arginine and l-lysine and these cells were separately incubated with either specific HRP-conjugated goat anti-chicken IgM or HRP-conjugated control antibody. Following biotinylation, equal numbers of specifically and nonspecifically biotinylated cells were mixed, lysed, and the biotinylated proteins from the pooled sample were isolated by streptavidin pull-down and TCEP elution. The experiment was repeated with reciprocal isotopic labeling. Quantitative data from 205 proteins present in both experimental replicates were analyzed (Fig. 3*A*, supplemental Table S2). For each replicate, those proteins with specific/nonspecific SILAC ratios that were greater than 1 S.D. from the median of the distribution were taken as highly significant. Twelve proteins matched this criterion. Nine were intrinsic plasma membrane proteins, and three were peripherally associated plasma membrane proteins. In six of these cases, there was prior literature evidence for a role in BCR signaling (Table 1).

**TABLE 1:** **Proteins with specific/nonspecific SILAC isotope ratios consistently greater than 1 S.D. above the median for both SILAC replicates**

Protein name	Localization	Previously implicated in BCR signaling?
Evi2a	Intrinsic plasma membrane	No
Na,K-ATPase β3 subunit	Intrinsic plasma membrane	Yes (62)
Na,K-ATPase α1subunit	Intrinsic plasma membrane	No
Raftlin	Intrinsic plasma membrane	Yes (14)
Integrin β1	Intrinsic plasma membrane	Yes (44)
Integrin α3	Intrinsic plasma membrane	No
PTPRC (CD45)	Intrinsic plasma membrane	Yes (71)
CD98LC (SLC7a5)	Intrinsic plasma membrane	No
chB6 (Bu-1)	Intrinsic plasma membrane	No
Guanine nucleotide-binding protein G(i) subunit α2 (GNAI2)	Peripheral plasma membrane	No
CDC42	Peripheral plasma membrane	Yes (30)
RhoA	Peripheral plasma membrane	Yes (55)

We used immunoprecipitation to confirm the biotinylation of the selected proteins in Table 1. We chose two proteins, for which appropriate species cross-reacting antibodies were available. These were chB6, an intrinsic plasma membrane protein with a prominent extracellular region (16), and CDC42, a cytosolic protein that is also peripherally associated with the plasma membrane (30). Both chB6 and CDC42 proteins were separately immunoprecipitated from cells that had been specifically and nonspecifically labeled. Only the proteins isolated from the specifically labeled cells were biotinylated, although the degree of biotinylation was considerably greater for chB6 (Fig. 3*B*). To retain the biotin label, we carried out SDS-PAGE separations without reducing agent (see “Experimental Procedures”). Under these conditions, the anti-chB6 antibody detected bands of about 140–150 and 70–75 kDa (Fig. 3*B*) in the immunoprecipitates. The protein chB6 is known to be a disulfide-linked homodimer, and the 140–150-kDa band has been previously identified as the dimeric species (31). Interestingly, only the 140–150-kDa band was biotinylated (Fig. 3*B*) (see “Discussion”).

Several proteins with SILAC ratios just below the median +1 S.D. level were components of the cytoskeleton, ribosomes, and the mitochondria (Fig. 3*A*, supplemental Table S2). Interestingly, mitochondria gathered underneath the BCR clusters in DT40 cells (Fig. 3*C*). This suggests that at least some proteins in this region of Fig. 3*A* may also convey biologically significant information (see “Discussion”).

##### Close Association of Cross-linked BCR with Raftlin and chB6

**FIGURE 4.**
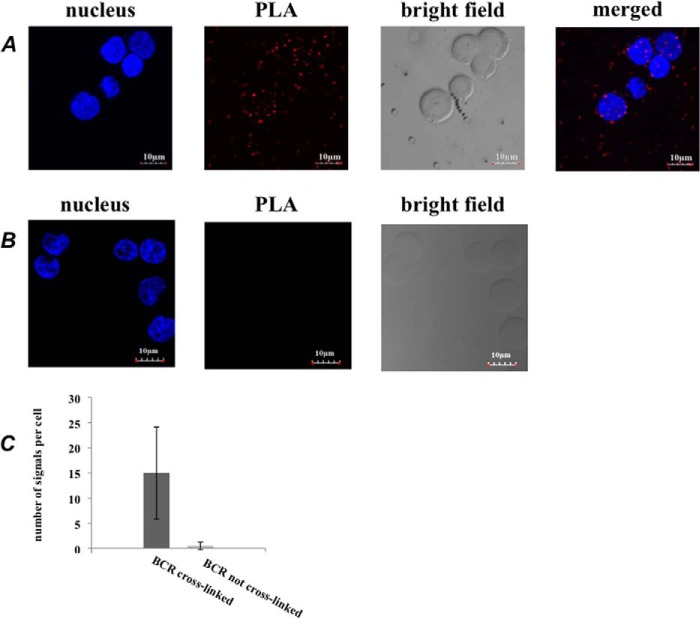
**BCR cross-linking drives the close association of BCR with raftlin.** *A*, PLA signal between raftlin and BCR in cells cross-linked with anti-BCR to induce BCR clustering. *Bar* = 10 μm. *B*, control cells fixed before anti-BCR addition showing no PLA stain in the absence of BCR clustering. *Bar* = 10 μm. *C*, quantification of data shown in *A* and *B*.

The cross-linked BCR enters lipid rafts (14, 32, 33). We therefore expect that SPPLAT should detect evidence of this dynamic association. We detected raftlin in our data set as one of the proteins with significant SILAC ratios (Table 1). To provide independent confirmation of this association, we performed a PLA between BCR and raftlin, both before and after BCR cross-linking. PLA is a very sensitive and stringent method to detect proximity between two proteins that are typically within 30–40 nm of each other (34). We detected a specific PLA signal between BCR and raftlin, but only after prior BCR cross-linking (Fig. 4). Hence, SPPLAT was able to confirm a known aspect of BCR behavior (2).

**FIGURE 5.**
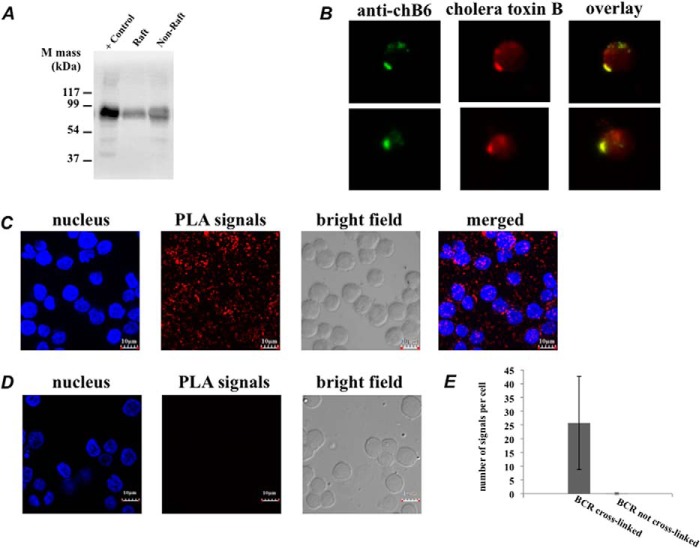
**The chicken allotypic marker chB6 partitions into lipid rafts and becomes associated with BCR following BCR cross-linking.** *A*, lipid raft and non-raft fractions were isolated from the DT40 plasma membrane (∼10^6^ cells), separated by SDS-PAGE under reducing conditions, and blotted for chB6 expression as described under “Experimental Procedures.” *B*, DT40 cells were incubated with anti-chB6 for 20 min on ice, and double stained with anti-mouse FITC and Alexa 594 cholera toxin B. Cells were visualized by epifluorescence microscopy. *C*, PLA signal between chB6 and BCR in cells cross-linked with anti-BCR to induce BCR clustering. *Bar* = 10 μm. *D*, control cells fixed before anti-BCR addition showing no PLA stain in the absence of BCR clustering. *Bar* = 10 μm. *E*, quantification of data shown in *C* and *D*.

We should also expect to detect other proteins that partition into lipid rafts following BCR cross-linking. As noted above, the protein chB6 consistently exhibited highly significant SILAC ratios and was selectively biotinylated (supplemental Table S2, Fig. 3*B*). This protein exhibits alloantigenic polymorphism, and has been used extensively as a marker of avian B-lymphocytes (35, 36), yet its functional significance is unknown. Subcellular fractionation and immunofluorescence co-localization with cholera B toxin confirmed that chB6 partitioned into the lipid raft fraction of the plasma membrane (Fig. 5, *A* and *B*). We therefore examined the co-localization of BCR and chB6 using PLA analysis. There was a strong PLA signal following BCR cross-linking. Moreover, the PLA signal was completely absent in control cells whose BCR had not been prior cross-linked (Fig. 5, *C–E*). This indicates there must be a significant rearrangement of chB6 following BCR cross-linking such that the two molecules are drawn together into very close proximity within the cluster.

##### Structural Insights into the chB6 Protein

**FIGURE 6.**
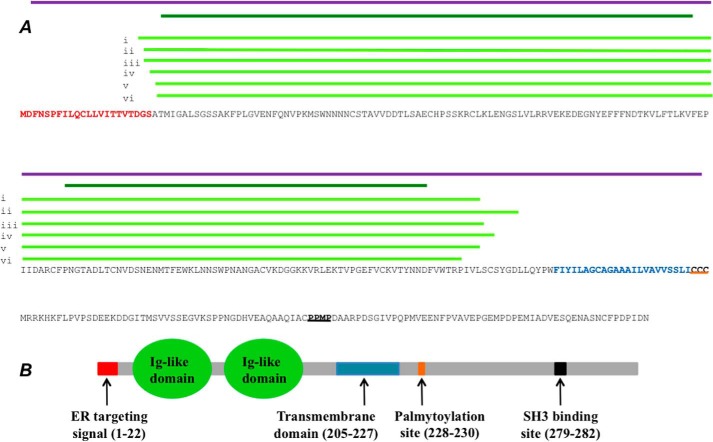
**Putative functional regions and regions of Ig domain sequence similarity identified in chB6.** *A,* relationship between amino acid sequence and putative functional regions. *Red sequence*: ER targeting signal. *Blue sequence*: transmembrane domain identified using SMART. *Orange underline*: putative palmitoylation site. *Black underline*: putative SH3-domain binding site. *Purple line*: region of sequence similarity to CD2 detected by PSI-BLAST. *Dark green line*: regions of sequence similarity to Ig domains detected by SMART. *Light green lines*: regions of sequence similarity to Ig domains detected with FUGUE. Specific proteins identified by FUGUE are: (i) growth arrest specific protein (*Z* score 16.23); (ii) junction adhesion molecule (*Z* score 15.31); (iii) high affinity Fc receptor (*Z* score 14.55); (iv) CD43 (*Z* score 14.34); (v) SLAMf6 (*Z* score 11.76); (vi) T lymphocyte activation antigen CD80. For further details of analysis methods see “Results.” *B*, schematic summary of putative functional regions in chB6.

The original description of chB6 indicated an intrinsic membrane protein, with a single transmembrane domain and a heavily glycosylated extracellular region. However, no sequence similarity to other proteins has previously been described (16). We re-examined the sequence of chB6 using more sensitive homology searches (Fig. 6). We first searched the chB6 sequence against the SMART database (version 7) (37). This analysis identified the endoplasmic reticulum targeting signal and a single transmembrane domain. Three membrane-proximal cysteines at positions 228–230 indicate a likely palmitoylation site (Fig. 6*A*). This feature is commonly found in proteins that are targeted to lipid rafts (38).

The intracellular sequence of chB6 contains conserved proline residues typical of SH3-domain binding sites that are found in signal-transducing receptors (39). Within the extracellular region, our analysis detected the presence of two immunoglobulin (Ig)-like domains (Fig. 6*A*). Using the iterative PSI-BLAST program (40), we identified putative chB6 orthologs in birds and turtles, but also more distantly related proteins. A consistent feature of all these proteins was the presence of two immunoglobulin domains within their extracellular regions (Fig. 6, *A* and *B*). Many of these proteins are members of the CD2 family of immune receptors. We next analyzed the chB6 sequence using the FUGUE sequence-structure homology recognition server. FUGUE takes an input sequence and compares it to a database containing known three-dimensional structures clustered into related families (41). Sequence alignments are identified using environment-specific substitution tables. The sensitivity of this approach relies on the fact that secondary and tertiary structures diverge more slowly from a common ancestor than primary sequences (42). With chB6, FUGUE again detected significant homologies to proteins that contain extracellular Ig domains (Fig. 6*A*). Hence, our data consistently places chB6 within the extended immunoglobulin superfamily (Igsf) of cell signaling/cell-adhesion molecules.

##### chB6 Cross-linking Activates Cell Binding to Integrin Substrates

**FIGURE 7.**
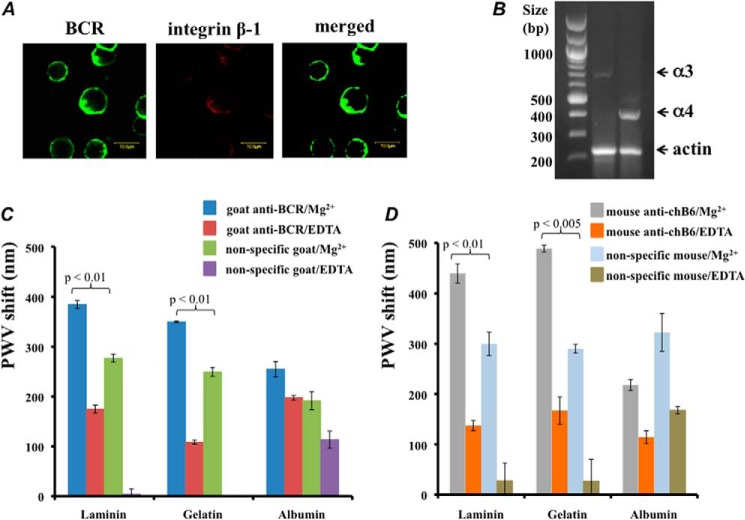
**Cross-linking the BCR and the chB6 alloantigen in DT40 cells stimulates integrin-mediated cell adhesion.** *A*, co-localization of cross-linked BCR with β1 integrin. *Bar* = 10 μm. *B*, RT-PCR for α3 integrin, α4 integrin, and actin. *C* and *D*, cell binding to the integrin substrates. Cells were preincubated with goat anti-chicken IgM or non-immune goat antibody (*C*), or with mouse anti-chB6 or non-immune mouse antibody (*D*), as described under “Experimental Procedures.” Cells were incubated with substrate-coated wells (laminin, gelatin, or albumin), and binding was assayed as the shift in peak wavelength value (*PWV*) after a 2-h incubation, using the SRU Biosystems BIND Explorer system. Values are mean ± S.D. of 3 replicates. The *p* values for experiments with and without antibody cross-linking are indicated.

Integrins are heterodimers containing one α and one β subunit selected from 18 α and 8 β subunits in 24 known pairings, forming a well characterized family of cell-adhesion molecules (43). In each of our reciprocal SPPLAT experiments, we obtained quantitative data for unique peptides corresponding to β1 and α3 integrin subunits (supplemental Table S2). We also detected co-localization of the integrin β1 subunit with the cross-linked BCR (Fig. 7*A*). Previous work has identified the integrin α4 subunit in DT40 cells (15), but not α3. As judged by RT-PCR, both integrin α3 and α4 subunits were expressed in DT40 cells, but with substantially more α4 (Fig. 7*B*). Although we detected α4 peptides, they did not give quantitative data in all experiments (supplemental Table S3).

In B-lymphocytes, including DT40, the cross-linked BCR activates integrin-dependent cell-binding (15, 44). Molecules such as CD2 act as “integrin regulators” that co-stimulate integrin-mediated cell binding during lymphocyte activation (45). In light of our data showing that chB6 may be a distant paralog of CD2-like molecules, we investigated integrin regulation by chB6. A convenient way to study this phenomenon is to induce integrin activation with appropriate cross-linking antibodies (46). Different integrin isoforms show distinct patterns of ligand specificity. Activated integrin α3β1 binds to laminin (47), and gelatin contains exposed RGD motifs that are recognized by many integrin isoforms (26). Using a real-time cell-binding assay, we confirmed that cross-linking chB6 on DT40 initiated cell binding to both laminin and gelatin. The chB6-induced binding was even stronger than that induced by BCR cross-linking, and as expected for an integrin-mediated reaction, it was magnesium-dependent (Fig. 7, *C* and *D*).

## DISCUSSION

Enzyme-generated proximity labeling is well established in histochemistry, where the method is used to provide localized signal amplification (48). An early proteomic application of this concept exploited the presence of endogenous peroxidases combined with fluorescently labeled tyramine (49). Recently, several groups have generalized the method to deliver the peroxidase onto a predetermined target. For example, coupling the enzyme to cholera toxin B facilitated the identification of lipid raft proteins (8). In another variation, cells have been generated that stably express peroxidase in mitochondria, enabling the proteomic analysis of this organelle (9). Alternatively, cells have been generated that express biotin ligase chimeras that enable the biotinylation of interacting partners (50).

For our assay, we chose tyramide as the labeling reagent because the activated reagent “footprint” is typically tens to a few hundreds of nanometers (51). Biotin was chosen because of its strong and specific affinity for streptavidin. The introduction of a spacer arm allows efficient affinity capture, and the disulfide bond enables high-yield specific recovery from the affinity column following reduction. These characteristics are well suited for the analysis of extended surface-localized protein assemblies, as exemplified by the BCR clusters.

A characteristic feature of the cross-linked BCR in B-lymphocytes is that proteins coalesce as large asymmetric patches within the plasma membrane (28, 52). This makes it easier to demonstrate the close co-localization of the BCR with deposited biotin (Fig. 2*A*). Immunofluorescence imaging indicated that the deposited biotin was largely restricted to the plasma membrane, and most biotin could be removed with a membrane impermeant reducing agent (Fig. 2*B*). Alternative arylazide proximity labels can cross the plasma membrane, and could generate high levels of nonspecific binding because they are activated by intracellular enzymes (8).

Although there have been proteomic studies of the T cell immune synapse (53), the molecular composition of the BCR clusters is not so well defined. To our knowledge, a proteomic study on purified total lipid rafts from human B-lymphocytes is the only such published example (54). But this work examined all lipid raft proteins, and was not specifically intended to identify proteins in close proximity to the BCR. Our work is the first attempt to apply the newly developed proximity labeling assays to the assembled BCR, and thereby identify co-localized proteins in this complex system.

Of the 12 highly significant proteins listed in Table 1, 9 are intrinsic plasma membrane proteins. However, 3 of the proteins are peripherally associated with the cytosolic face of the plasma membrane. These are RhoA, CDC42, and guainine nucleotide-binding protein G(i) subunit α2 (GNAI2). RhoA and CDC42 are GTP-binding proteins that have been previously implicated in the regulation of BCR clustering (30, 55, 56). In DT40, the protein Vav3 acts as a guanine nucleotide exchange protein for this family of G proteins. Deletion of Vav3 in DT40 compromises BCR signaling, but expression of constitutively active cdc42 and RhoA can re-activate downstream signaling events in these cells (57).

As judged by immunofluorescence, most of the tyramide-biotin was deposited onto the external face of the plasma membrane (Fig. 2*B*). Nevertheless, some of the label can evidently cross the membrane and biotinylate peripherally associated proteins such as CDC42. However, the degree of CDC42 biotinylation was clearly much less than that displayed by the intrinsic membrane protein chB6 (Fig. 3*B*). It is possible that CDC42 lies at the extremity of the biotinylation footprint emanating from the extracellularly bound HRP-conjugated anti-IgM antibody. It is also possible that such peripheral proteins, although poorly biotinylated themselves, show significant SILAC ratios because they interact with other biotinylated membrane proteins within the cluster, and these interactions survive purification on the strepatavidin column.

It should be noted that the conceptually similar proximity labeling assay that targeted lipid raft components, mentioned above, also detected cytosolic peripherally associated plasma-membrane proteins (8). Indeed, one of the peripheral proteins identified in this assay was GNAI2, which we also detected in our significant data set (Table 1). Our results indicate a close association of the cross-linked BCR with raftlin (Fig. 4, supplemental Table S2), and the cross-linked BCR is known to enter lipid rafts (14). Hence our data provide additional support for the view that GNAI2 is a marker for lipid rafts (8).

We emphasize that proteins detected by proximity labeling assays need not interact with the target protein directly, but merely lie within a limited distance from the target. We therefore suggest that proximity labeling assays such as SPPLAT will be particularly appropriate for the analysis of dense but localized membrane-bound protein clusters. The BCR model studied here is a good example, as are lipid rafts (8) and proteins tightly restricted within intracellular compartments, such as mitochondria (9).

**FIGURE 8.**
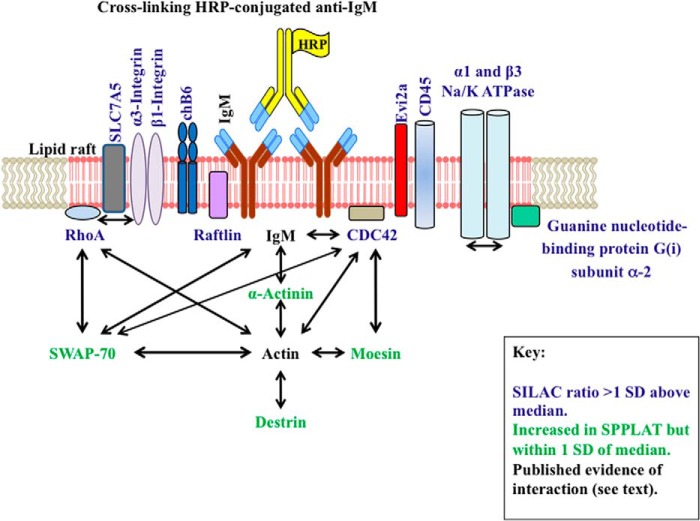
**Summary of the key proteins identified by SPPLAT.** Proteins with specific/nonspecific SILAC isotope ratios consistently greater than 1 S.D. above the median for both SILAC replicates are indicated as schematics, and labeled in *blue*. Additional key cytosolic proteins identified by SPPLAT and discussed in the text are indicated in *green. Black arrows* indicate known interactions between proteins.

Given the relative novelty of such proximity assays, one aspect of this work was to gain practical insight into their application. Our experience suggests that these assays will require some form of proteomic quantitation if we are to effectively separate promising candidates from proteins that bind nonspecifically to the affinity matrix. In particular, quantitation was useful to discern different degrees of likely biological significance. For example, in our data set, there was a notable separation of distinct organelle proteins. In particular, nuclear proteins were predominantly found with low SILAC ratios, mostly below the median (Fig. 3*A*). This is consistent with the nuclear proteins being nonspecific contaminants, which is likely to be a particularly prominent feature with DT40, given the large nuclear to cytoplasmic ratio of these cells (10). Mitochondrial, ribosomal, and some cytoskeletal proteins typically displayed rather higher SILAC ratios, mostly within 1 S.D. of the median, and distinct from many of the nuclear proteins (Fig. 3*A*). Interestingly, there was a clear accumulation of mitochondria underneath the BCR clusters in DT40 cells (Fig. 3*C*). Mitochondria are known to accrue under the immune synapses of T cells (53). However, as far as we are aware, the concentration of mitochondria underneath B-lymphocyte BCR clusters has not been previously noted. The cytoskeletal regulators switch-associated protein 70 (SWAP-70), moesin, and destrin were also present in this region of the SILAC plot (supplemental Table S2). They are known to accumulate underneath the cross-linked BCR, where they play important roles in coordinating BCR assembly (58). Ribosomes similarly concentrate under the T cell immune synapse (53). As noted above, the biotin was largely deposited on the surface of the cells (Fig. 2*B*), although a modest degree of biotinylation could be detected for proteins closely associated with the BCR but located on the cytoplasmic face of the plasma membrane (Fig. 3*B*). Thus we suggest that these intracellular proteins display slightly elevated SILAC ratios not because they are strongly biotinylated themselves, but because they are connected to biotinylated surface proteins via multiple low affinity, but high avidity interactions within the cytoskeletal matrix (58). A summary of these interactions, with one another and with plasma membrane proteins identified by SPPLAT is given in Fig. 8.

The assembly of the BCR cluster is dynamic, and its composition may change during the cross-linking process. Based on the successful identification of proteins known to be functionally associated with the BCR, we conclude that our time frame was physiologically relevant. Our current protocol may, however, lead to under labeling of some molecules. For example, the BCR is composed of an IgM antibody together with auxiliary subunits Ig-α and Ig-β (52). Antigen-induced cross-linking of IgM leads to the dissociation of both of these subunits from the BCR (59). We did selectively detect Ig-β in our BCR immunoprecipitation (supplemental Table S1), and we did recover Ig-β in our SILAC data set, but with too few peptides for accurate quantitation in all samples (supplemental Table S3). It is possible that there had been partial dissociation of these subunits prior to the tyramide-labeling step under the conditions we used.

The BCR itself displayed a relatively low SILAC ratio (supplemental Table S2). In preliminary Western blotting experiments, we detected significantly more BCR in streptavidin-isolated proteins from specifically labeled cells compared with nonspecifically labeled cells (data not shown). Furthermore, MS analysis confirmed that the BCR was immunoprecipitated by anti-chicken IgM (supplemental Table S1). This suggests that the relatively low SILAC ratio of the BCR may not reflect the full extent of enrichment in the selectively biotinylated fraction. The BCRs can self-assemble in lysed samples *in vitro* (60), and it is possible that post-lysis mixing of heavy and light isotope-labeled BCRs may be occurring in this case. Post-lysis mixing is a known phenomenon that can artifactually reduce SILAC ratios (61).

Among the proteins with significant SILAC ratios, a surprising finding was the detection of α1 and β3 subunits of the Na,K-ATPase. However, a monoclonal antibody specific for the β3 subunit of the Na,K-ATPase inhibits mitogen activation of both T- and B-lymphocytes (62). Another interesting protein identified by SPPLAT was Evi2a. Very little is known about the function of this protein. Homology searches using PSI-BLAST, SMART, and FUGUE all failed to identify any features conserved with other proteins. In mice, several leukemogenic retroviruses integrate close to the *Evi2a* gene in lymphocytes and disrupt its expression (63). Hence, it is possible that Evi2a is a lymphocyte-specific tumor suppressor. Our results provide the first indication that Evi2a could be involved in BCR function.

The protein chB6 (Table 1, Figs. 3*B* and 5) is particularly interesting. It is used extensively as an allotypic B-lymphocyte marker in avian immunology (35, 36). Despite the fact that its cDNA was cloned and sequenced nearly 20 years ago (16), there is still very little known about its structure and function. It has been suggested that chB6 is a “death receptor,” and a phylogenic analysis has claimed that chB6 is related to TNFR-2 (64). However, we do not find any statistically significant sequence similarity at the amino acid level between chB6 and TNFR-2. Furthermore, chB6 lacks other expected features of death receptors such as intracellular death domains (35). In contrast, our sequence analysis indicates that chB6 is a type I membrane protein with a single transmembrane domain and two Ig-like domains in the extracellular region (Fig. 6). It is related to a large class of vertebrate Ig domain-containing immune system proteins that include CD2-like proteins (65) and the SLAM family of immune regulators (66). Interestingly, the gene encoding chB6 lies within 20 kb of the *CD2* gene on chicken chromosome 1. Other genes encoding Ig domain immune regulators are also very closely linked within this chromosomal region including CD80, a protein identified by our FUGUE search (Fig. 6*A*) (ncbi.nlm.nih.gov/gene?term = X92867).

The Western blot of immunoprecipitated chB6, analyzed by non-reducing SDS-PAGE displayed bands at 140–150 and 70–75 kDa (Fig. 3*B*). The higher molecular weight band was not seen under reducing SDS conditions (Fig. 5*A*). Hence, chB6 most likely forms a disulfide-linked homodimer in DT40 cells. This property of chB6 has been noted previously (31). There are eight cysteine residues within the chB6 extracellular region (Fig. 6*A*). Four of these cysteines will most likely form intramolecular disulfide bonds, one intramolecular disulfide bond for each Ig domain (67). This leaves up to four cysteines that could potentially form intermolecular disulfide bonds. Only the higher molecular weight form of chB6 was biotinylated (Fig. 3*B*). This is consistent with the demonstration that the disulfide-bonded dimer is present on the lymphocyte plasma membrane (31). The prominent non-biotinylated 70–75-kDa species detected in the immunoprecipitations (Fig. 3*B*) probably represents the immature form of the protein localized within the intracellular secretory pathway. The dimeric structure of chB6, together with its close association with the cross-linked BCR could induce longer range cross-linking, and so enhance cluster stability on the surface of the membrane.

Members of the CD2 family can regulate integrin-mediated cell binding (46), and we observed enhanced cell binding to the integrin substrates laminin and gelatin following cross-linking with anti-chB6 antibodies. Indeed, this effect was slightly stronger than the integrin activation induced by cross-linked BCR. Nonspecific antibodies of the appropriate species induced significantly less cell binding (Fig. 7, *C* and *D*).

The only integrin peptides with significant SILAC ratios and quantified in every replicate corresponded to the α3 and β1 isoforms. Laminin is the selective ligand for integrin α3β1 (47) and is a major component of the basal lamina, where it may support neoplastic B cell extravasation (68). The protein SLC7A5 (CD98LC) (Table 1) associates with integrin α3β1 (69). Similarly, CD45 (Table 1) can regulate integrin-mediated lymphocyte cell adhesion (70). Deletion of the *CD45* gene in DT40 cells has been shown to compromise BCR signaling (71). Interestingly, all these interactions take place in lipid rafts (2, 69, 72).

In DT40 cells, cross-linking the BCR, and cross-linking chB6 both induce apoptosis when conducted in solution (12, 31). However, integrin binding to its substrates is known to inhibit apoptotic signaling (44). The integrin regulation controlled by proteins in the BCR cluster may ensure the selective survival of correctly adhered antigen-stimulated cells. Our work thus places chB6 into its broader evolutionary context and is the first to link chB6 both with the BCR and with integrin-mediated cell binding. The chB6 protein is a good example of the sort of molecule we hoped and expected to find using our SPPLAT approach. In conclusion, our proximity labeling approach has yielded novel targets for the study of B cell biology, and should have broad applications within contemporary cell biology.

## Supplementary Material

Supplemental Data

## Footnotes

6 The abbreviations used are:

BCR: B cell receptor
GNAI2: guainine nucleotide-binding protein G(i) subunit α2
SILAC: stable isotope labeling by amino acids in cell culture
SPPLAT: selective proteomic proximity labeling assay using tyramide
TCEP: Tris(2-carboxyethyl)phosphine hydrochloride
PLA: proximity ligation assay.
