# Supplementary material for: New Insights into the DT40 B Cell Receptor Cluster Using a Proteomic Proximity Labeling Assay
Source: J Biol Chem. 2014 Apr 4;289(21):14434–47. doi: 10.1074/jbc.M113.529578 (PMC4031500; doi:10.1074/jbc.M113.529578)
Supplement: Supplemental Data [file supp_289_21_14434__index.html]

New Insights into the DT40 B-Cell Receptor Cluster using a Proteomic Proximity Labeling Assay — New Insights into the DT40 B Cell Receptor Cluster Using a Proteomic Proximity Labeling Assay — Proteomic Study of Localized B Cell Receptor Surface Clusters — Supplemental Data 

# New Insights into the DT40 B Cell Receptor Cluster Using a Proteomic Proximity Labeling Assay

## Supplemental Data

**Files in this Data Supplement:**

- Supplemental Table 1 (.xlsx, 114 KB) - Proteins identified by immunoprecipitation using anti-(chicken IgM) and non-specific antibody control.
- Supplemental Table 2 (.xlsx, 34 KB) - Proteins identified and quantified in both SILAC experiments.
- Supplemental Table 3 (.xlsx, 14 KB) - Summary of integrin and Ig&#x26;#946; peptides identified in SILAC experiments.
